# Antiplatelet agents for cancer treatment: a real perspective or just an echo from the past?

**DOI:** 10.1007/s10555-017-9683-z

**Published:** 2017-07-27

**Authors:** Marek Z. Wojtukiewicz, Dominika Hempel, Ewa Sierko, Stephanie C. Tucker, Kenneth V. Honn

**Affiliations:** 1 Department of Oncology, Medical University of Bialystok, 12 Ogrodowa St., 15-025, Bialystok, Poland; 2 Department of Radiotherapy, Comprehensive Cancer Center in Bialystok, Bialystok, Poland; 3 Department of Clinical Oncology, Comprehensive Cancer Center in Bialystok, Bialystok, Poland; 4 Department of Pathology-School of Medicine, Bioactive Lipids Research Program, Detroit, MI 48202 USA; 5 Departments of Chemistry, Wayne State University, Detroit, MI 48202 USA; 6 Department of Oncology, Karmanos Cancer Institute, Detroit, MI 48202 USA

**Keywords:** Platelet, Cancer, Anti-platelet therapy

## Abstract

The association between coagulation and cancer development has been observed for centuries. However, the connection between inflammation and malignancy is also well-recognized. The plethora of evidence indicates that among multiple hemostasis components, platelets play major roles in cancer progression by providing surface and granular contents for several interactions as well as behaving like immune cells. Therefore, the anticancer potential of anti-platelet therapy has been intensively investigated for many years. Anti-platelet agents may prevent cancer, decrease tumor growth, and metastatic potential, as well as improve survival of cancer patients. On the other hand, there are suggestions that antiplatelet treatment may promote solid tumor development in a phenomenon described as “cancers follow bleeding.” The controversies around antiplatelet agents justify insight into the subject to establish what, if any, role platelet-directed therapy has in the continuum of anticancer management.

## Introduction

The idea that platelets contribute to tumor growth and progression is not new, and multiple mechanisms have been described that explain these complex platelet/tumor cell interactions [1–3]. Advanced knowledge about the molecular and functional aspects of platelet-mediated tumor dissemination motivated scientists to search for drugs with anticancer potential. The experimental models demonstrated that inhibitors of platelet-evoked processes as well as thrombocytopenia reduce tumor metastasis, while clinical data indicate that anticoagulant (AC) therapy may influence cancer-specific mortality [4–6].

The most important processes contributing to cancer progression regulated by platelets and their granular contents are adhesion and tumor cell-induced platelet aggregation (TCIPA), which result in tumor emboli in microvasculature, tumor cell migration, intra-/extravasation and angiogenesis [3, 7]. Additionally, platelets are critical for survival of tumor cells within solid mass as they regulate intratumoral vasculature and hemostasis, and they protect circulating tumor cells (CTCs) in the blood stream from NK cell lysis [8–10]. Moreover, platelets are able to recognize pathogens and secrete active cytokines and so should be regarded as an important component of the immune system [11].

**Fig. 1:**
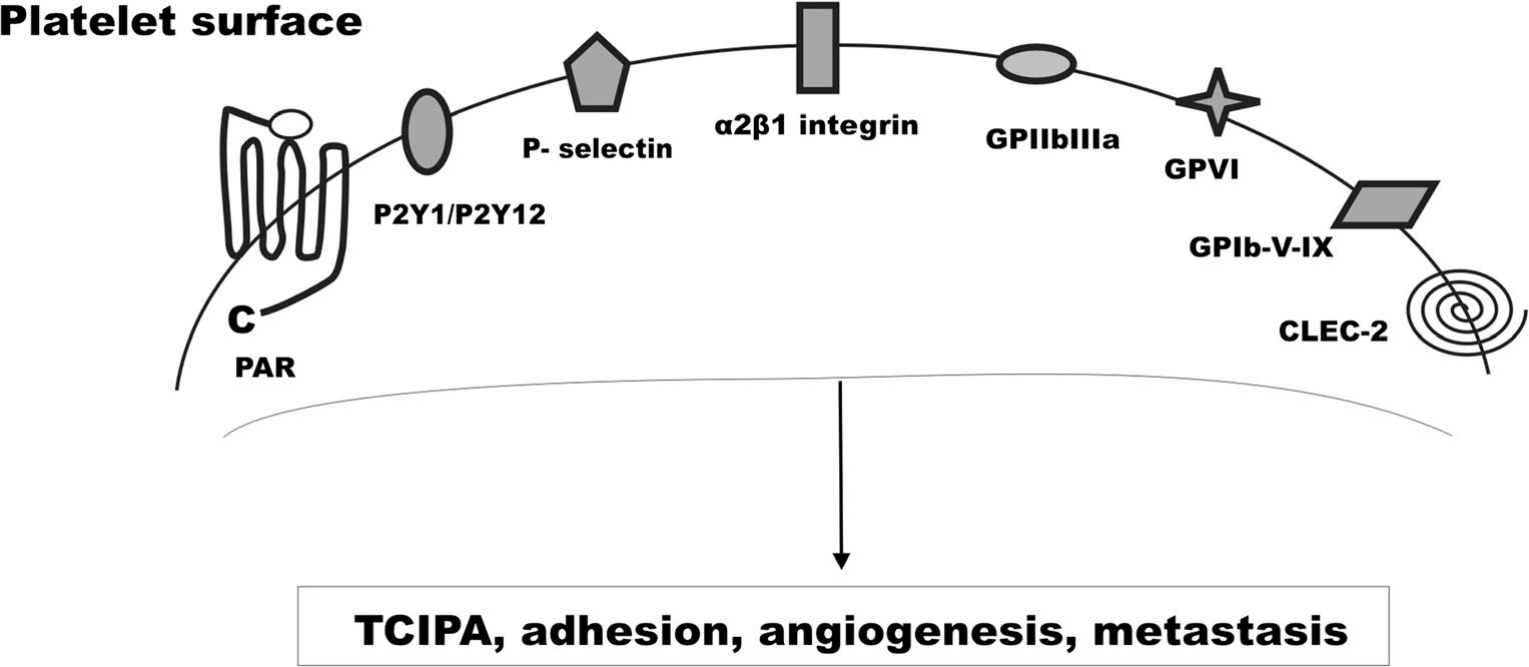
Platelet receptors and molecules contributed to cancer dissemination. *CLEC-2* C-type lectin-like receptor-2, *GPIb-IX-V* glycoproteins Ib-IX-V, VI, IIb-IIIa, *PAR* protease-activated receptors for thrombin, *P2Y* purigenic P2 receptors for nucleotides, *TCIPA* tumor cell-induced platelet aggregation

Platelet activation pathways are associated mainly with their membrane receptors, which may be activated during TCIPA and inhibited by their respective inhibitors (Fig. 1) [1, 12]. Platelet membranes contain integrins (e.g., glycoproteins Ib-IX-V (GP Ib-IX-V), glycoprotein VI (GP VI), and glycoprotein IIb-IIIa (GP IIb-IIIa), integrin αIIbβ3), thromboxane (TxA2) receptor (TPR), purigenic P2 receptors for nucleotides (adenosine diphosphate (ADT) and adenosine triphosphate (ATP)) as well as protease-activated receptors for thrombin (PAR-1 and PAR-4) that are intimately involved in cancer progression [13]. The newly discovered C-type lectin-like receptor-2 (CLEC-2 receptor) and its activator, podoplanin, a key protein in platelet aggregation, have attracted the interest of the scientific community [14, 15]. Additional attractive targets for antiplatelet treatment involve arachidonic acid (AA) metabolism, cyclooxygenase (COX) and lipoxygenase (LOX) enzymes, and many other compounds exhibiting antiplatelet activity [16].

Aspirin is an antiplatelet drug whose anticancer activity has been thoroughly investigated both *in vitro* and *in vivo* using cancer cell lines, animal models as well as clinical trials [17–19]. Targeting platelet cyclooxygenases (COX-1, COX-2) with low-dose aspirin exerts antimetastatic and antiproliferative effects [18, 19], and *in vivo* analyses indicate that aspirin may even reduce distant metastases rates (DMR), disease-free survival (DFS), and/or overall survival in cancer patients [20–24]. Moreover, a population-based historical cohort study and randomized trials have shown that aspirin prevents cancer incidence [25, 26]. New trials are underway [27]. Aspirin derivatives with less gastrointestinal effects are currently being investigated as therapeutics as well [28].

There is new and somewhat alarming data that prolonged anti-platelet/coagulant treatment may promote cancer development [29–31]. This effect has been described for prasugrel (TRITON trial), vorapaxar (TRACER trial, Thrombin Receptor Antagonist for Clinical Event Reduction in Acute Coronary Syndrome), and for 30-month therapy with prasugrel and clopidogrel (Dual Antiplatelet Therapy, DAPT trial). Long-term antiplatelet therapy was associated with cancer development that contributed to about half of the noncardiovascular deaths (NCVD) in these trials. However, the population-based cohort study among colorectal, breast, and prostate cancer patients did not confirm an increased risk of cancer-specific mortality in this group of patients using clopidogrel [32]. Obviously, targeting platelet receptors is an approach that can be associated with other serious side effects related to suppression of prothrombotic pathways such as an increased risk for bleeds in treated patients.

To dispel concerns and address controversies surrounding antiplatelet therapy, we have gathered the newest data on platelet receptors and focused on potential modes of inhibition (Fig.1).

## Markers of platelet activation in cancer patients

### Thrombocytosis

An increase in platelet number (thrombocytosis) and activity is seen in patients with malignancies and was first noticed by Reiss et al. in 1872 [33, 34]. Moreover, a large number of platelets are found in the tumor microenvironment outside of the blood vessels inducing angiogenesis and facilitating cancer cell dissemination [35]. The concept of using an antiplatelet approach for cancer treatment originated in 1968 when Gasic et al. [36] demonstrated that intravenous injection of neuraminidase resulting in thrombocytopenia was associated with decreased metastasis in a mouse model. Thrombocytosis is a poor prognostic indicator for epithelial ovarian carcinoma [37]. Further experiments on animal models and analysis in cancer patients confirmed these associations [38–42]. Moreover, platelet transfusion in orthotopic models of human ovarian cancer resulted in significantly greater tumor weight than in untreated mice or platelet-depleted mice, where in the latter condition, mean tumor weight was diminished by 70% [43]. Lower platelet counts and antiplatelet therapy independently predicted better outcomes in patients with head and neck squamous cell carcinoma, invasive ductal breast carcinoma, gastric cancer, and renal cancer [39, 40, 44]. Patients with rectal adenocarcinoma presenting lower platelet counts were more likely to answer with good or complete response to neo-adjuvant treatment than patients with higher platelet counts [41]. Similarly, reduced pre-operative platelet levels related to better histopathological features and improved overall survival in gastric cancer patients [41].

Recent data support the prognostic value of the platelet-lymphocyte ratio (PLR) in cancer patients (e.g. gastric, colorectal, esophageal, ovarian, lung cancer) [45–47]. Namely high peripheral blood PLR was related to poor tumor differentiation, local staging (T – tumor), recurrence of the disease, and decreased overall survival. Therefore, PLR may be used as a predictor of overall survival (OS) in association with clinicopathological parameters in cancer patients.

Platelet activation is observed in cancer patients and is detected by increased secretion of platelet content and release of microparticles and exosomes [2]. Biomarkers of platelet activation in cancer patients are soluble P-selectin, thrombospondin 1, TSP-1, β-thromboglobulin, CD40 ligand, transforming growth factor-β (TGF-β), platelet-derived growth factor (PDGF), vascular endothelial growth factor (VEGF), angiopoetin-1 (AP-1), matrix proteins, CCL17, CCXL1, CXCL5, and others [2, 48, 49]. Among the proteins identified on platelet exosomes is GAPDH, which can function as a membrane fusion protein and can serve as a plasmin/ogen receptor along with histone H2B that is also found on exosomes of different cell types [50–53]. This represents an underexplored source of immune suppression, as plasmin can potentially suppress NK cells through the release of fibrin and excessive exosome release could exhaust an NK response [10].

Seminal *in vitro* and *in vivo* studies have demonstrated that platelet activation contributes to the metastatic potential of cancer cells [1–3]. Intriguingly, the level of platelet reactivity may also contribute to prognosis of cancer patients [54]. The observation that decreased platelet reactivity, as measured by platelet surface expression of P-selectin, related to high risk of death in patients with cancer is most likely a consequence of continuous platelet activation.

It should be noted that there are no prospective studies assessing cancer incidence in healthy individuals with elevated platelet levels *versus* normal platelet levels. The results from mouse animal models where large numbers of malignant cells are injected directly into circulation cannot explain all aspects of cancer dissemination, which is a complex and multi-signal process in the human body. The elevated number of platelets in some cancer patients may be simply a paraneoplastic syndrome aroused by growth factors secreted by tumor cells.

### PMPs

Circulating tumor cells (e.g., of gastric, ovarian, oral, and prostate cancer) may activate platelets resulting in a production of small microparticles (MPs) that contain membrane receptors as well as cytoplasmic constituents [55–57]. Platelet-derived microparticles (PMPs) play a role in tumor growth and invasiveness (at least in part by stimulation of MMP-2 production), and angiogenesis [by releasing vascular endothelial growth factor (VEGF) and basic fibroblast growth factor (bFGF)], as well as induce metastasis [58]. The level of PMPs is an indicator for patient survival [59].

### TCIPA

The effect of platelet activation induced by tumor cells is associated with platelet aggregation (TCIPA) mediated by multiple agents: tissue factor (TF), thrombin, adenosine diphosphate (ADP), TxA2, and matrix metalloproteinases (MMPs) [60, 61]. Additionally, factors secreted by activated platelets elicit signaling within tumor cells and the tumor microenvironment so that the activation of cancer cells and platelets is reciprocal [62]. TCIPA is regarded as crucial for cancer dissemination. Studies based on three-dimensional visualization demonstrated clearly that tumor cells arrest in the pulmonary vasculature of mice in a complex with platelets and fibrinogen [63]. Tumor cell-platelet interactions in the pulmonary microvasculature were also studied *in vivo* following tail vein injection of murine Lewis lung carcinoma (LLC), 16C mammary adenocarcinoma, and B16 amelanotic melanoma tumor cells [64].

Importantly, tumor cells can be found surrounded by platelets in blood stream, which may afford protection from lysis by natural killer cells (NKs), demonstrating that TCIPA increases CTC survival. Moreover, activation of platelet-derived TGF-β decreases immunoreceptor NKG2D expression on the surface of NKs thus inhibiting their antitumor reactivity [65]. There is ample evidence that some antiplatelet drugs exert their antimetastatic effects by TCIPA inhibition (presented later).

## Platelet-associated molecules and therapy

### AA metabolites, COX, and LOX inhibitors

It is widely recognized that inflammation may trigger cancer initiation and progression [66–69]. Arachidonic acid is metabolized enzymatically *via* COX and LOX generating bioactive lipids termed eicosanoids: 2-series prostaglandins (PGs) and thromboxanes (Txs) (COX pathway) or 4-series leukotrienes (LTs) and hydroxyeicosatetraenoic acids (HETEs) (LOX pathway) [70–72]. Tumor cell-platelet interaction may activate COX individually or COX and LOX in concert resulting in production of the above mentioned eicosanoids. These in turn are involved in the growth and progression of cancers by regulating such processes as apoptosis, angiogenesis, and tumor cell invasiveness [68–73]. Thrombin-activated platelets (activation dependent on PARs) are a source of 12- and 15-HETE, as well as TxA2 [2, 74, 75]. The release of TxA2 into the microenvironment activates prostanoid receptors (TP) on platelets resulting in increased expression of GPIIb/IIIa (integrin αIIbβ3) and platelet aggregation. Preincubation of platelet-rich plasma with TxA2 receptor inhibitor SQ-29,548 decreased platelet aggregation induced by tumor osteogenic sarcoma cells without affecting TxA2 release [76]. TCIPA in carcinosarcoma cells resulted in 12-HETE activation and a subsequent increase in platelet expression of GPIIb/IIIa [77].

Due to the activities of COX and LOX pro-inflammatory metabolites, these enzymes are relevant targets for anticancer therapies [68–73].

#### Cyclooxygenase inhibitors

Compelling evidence exists for antitumor activity of the COX-1 and -2 inhibitor, aspirin, which disrupts platelet function by inhibiting prostaglandin and thromboxane formation [78–80]. Expression of COX-1 and/or COX-2 is cell-dependent. ECs and tumor cells express COX-2, but platelets express COX-1 [11]. It is difficult to distinguish which effects observed in experimental studies are attributable to which isoform of the enzyme, and the exact anticancer mechanisms of aspirin remain unexplained. There were hypotheses that aspirin benefits from COX-2 inhibition were from its antiproliferative and proapoptosis potential. However, it has been argued that aspirin at low doses could not result in the blood concentrations necessary to impact those processes [17]. Thus, the hypothesis that a dominant mechanism for the cancer preventive and anti-metastasis property of aspirin is associated with its antiplatelet activity (COX-1 inhibition) is plausible [78]. Platelets have been proven to impact metastases by direct and indirect interactions with tumor cells [1–3]. As aspirin can induce platelet apoptosis *via* caspase-3 activation [81], its cancer preventive role may be explained by thus interfering with platelet-induced epigenetic modifications of tumor cells during early phases of colorectal tumorigenesis, for example. Platelet activation in intestine may lead to local inflammation and cellular transformation [82].

Experiments with cancer cell lines *in vitro* provided proof that the anticancer effect of aspirin is associated with its influence on cross-talk between platelets and cancer cells (Table 1). Incubation of HT29 human colon carcinoma cells with human platelets induced their transformation from epithelial to mesenchymal-like features of cancer cells that is an indispensable step for cancer dissemination [3, 13]. Enhanced cancer cell mobility was the result of E-cadherin downregulation and Twist1 upregulation. There was also a proaggregatory effect on the platelets by the cancer cells. Similar results have been described for ovarian cancer cell line SK-OV-3, where platelets increased their invasive potential by inducing the epithelial-to-mesenchymal transition phenotype [85], but that could be inhibited by aspirin. The anticancer potential of aspirin when administered in low, antiplatelet doses to colon and pancreatic cancer cells was associated with inhibition of oncoprotein c-MYC expression and a reduction in proliferative potential of cancer cells [19].

**Table 1:** Aspirin role in cancer prevention and treatment—*in vitro and in vivo* studies

*In vitro* and *in vivo* studies	Effect
Zhao 2013 [81]	Platelet apoptosis *via* caspase-3 activation
Ding 2014 [83] and Ding 2017 [84] MM1.S and RPMI-8226 myeloma cell lines	Activation of caspases, upregulation of Bax, and downregulation of Bcl-2 and VEGF Potentiation of inhibitory effect of bortezomib
Cook 2015 [85] SK-OV-3 ovarian cancer cells	Inhibition of epithelial-to-mesenchymal transition phenotype
Mitrugno 2017 [19] SW480 colon and PANC-1 cancer cells	Inhibition of oncoprotein c-MYC expression and reduced proliferative potential of cancer cells
Vad 2014 [86] B16-F0 melanoma cells and skin B16-F0 melanoma tumor mouse model	Increase in reactive oxygen species (ROS) formation, Inhibition of tumor growth
Guillem-Llobat 2016 [18] HT29 human colon carcinoma cells	Inhibition of epithelial-to-mesenchymal transition phenotype reduced metastases rate
Sitia 2014 [87] Hepatocellular carcinoma	Reduction of immune-mediated pathological effects in the liver and tumorigenesis
Ogawa 2014 [66] Lewis lung carcinoma cells in mice model	Reduction of mediastinal lymph node metastasis
Etulain 2013 [88]	Inhibition of pro-angiogenic activity of the platelets
He 2017 [89] PC-3 prostate cancer cells	Induction of apoptosis by strong inhibition of NF-κB and decreases in the levels of phospho-Stat3 and phospho-Erk1/2

*VEGF* vascular endothelial growth factor, *NF-κB* nuclear factor-κB

The injection of platelet-exposed HT29 cells into the tail vein of humanized, immunodeficient mice resulted in higher incidence of lung metastasis. Inhibition of platelet COX-1 by aspirin prevented the *in vitro* changes as well as reduced the metastatic rate *in vivo* [1, 17]. The inhibitory effect of aspirin was also observed on B16-F0 melanoma cell proliferation and tumor growth in a mouse model [86].

Preclinical experiments also demonstrated the vital role of platelet activation in pathogenesis of hepatitis B virus (HBV)-associated hepatocellular carcinoma (HCC) as well as the anticancer effectiveness of dual platelet inhibitors (aspirin plus clopidogrel) [87, 90]. Platelet activation in HBV-HCC mouse models led to accumulation of virus-specific CD8 T cells and virus-non-specific inflammatory cells in the liver, increased hepatocellular proliferation, liver fibrosis, and hepatocarcinogenesis. Platelet inhibition prevented injury of liver cells and tumor development most likely due to the reduction of immune-mediated pathological effects in the liver.

Aspirin has also been shown to reduce mediastinal lymph node metastasis in a mouse model after injection of LLC cells [66]. Aspirin had no effect on primary tumor in the lung but significantly reduced mouse mortality. There is heterogeneity in the human response to aspirin in the setting of lung cancer. An English study found no survival benefit [91], while a German study suggested that there are benefits to specific subgroups that warrant careful scrutiny [92].

Aspirin is also an important antiangiogenic agent [88]. Human platelets activated with thrombin secrete both VEGF and endostatin, ultimately promoting tubule-like formation and increased proliferation of endothelial cells. Surprisingly, these proangiogenic actions were only slightly suppressed by VEGF receptor-neutralizing antibody. The other platelet-induced signaling inhibitors of protein kinase C (PKC), p38, ERK1/2, Src kinases, or PI3K/Akt also exerted only partial inhibitory effects. In contrast, aspirin fully blocked the pro-angiogenic activity of the platelet releasate.

A novel derivative of aspirin, nitric oxide- and hydrogen sulfide-releasing hybrid or NOSH-aspirin (NBS-1120), has been developed that is stronger than classic aspirin in its potential to inhibit tumor growth and decrease tumor size [28]. Moreover, it is suggested that aspirin does not prevent TCIPA while the aspirin prodrug, 5-nicotinate salicylate (ST0702 salicylate), is effective in TCIPA inhibition [93].

Another inhibitor of platelet COX and PDGF, trapidil, does prevent spontaneous pulmonary metastases formation in mice, but only when administered after excision of the primary tumor [94]. Non-steroidal anti-inflammatory drugs, e.g., flurbiprofen, prostacyclin analogues, and thromboxane A2 synthase inhibitors, also significantly decrease spontaneous metastases formation [95, 96].

An antagonist of the prostaglandin(PG)E2 EP3 receptor, DG-041, prevented induction of mesenchymal-like changes in colon cancer cells by lowering E-cadherin expression and elevating Twist1 protein resulting in disrupted metastatic potential. Cancer cell motility and proaggregatory action on platelets were also abrogated by this drug [18].

Solid-phase von Willebrand Factor (sVWF) is known to mediate TCIPA. Unstimulated platelets can adhere irreversibly to sVWF and stimulation by ADP, thrombin, or ristocetin increases adherence [97]. However, aggregation can be blocked by S-nitroso-glutathione (GSNO) and prostacyclin (PGI(2)). Moreover, pre-incubation of platelets with PGI(2) inhibits sVWF-tumor cell-stimulated platelet surface expression of GPIIb/IIIa essential for tumor cell/platelet interactions [98].

The majority of clinical data relates to aspirin use in colorectal cancer (CRC) which is well recognized to be associated with inflammation (colitis–dysplasia–carcinoma) [78]. Although the Physicians’ Health Study reported in 1998 that they found no association between the use of aspirin and the incidence of CRC [99], subsequent studies reported more promising results [24, 100]. Long-term effects of aspirin on CRC incidence and mortality during the more than 20-year follow-up were presented in the analysis of their randomized trials [24, 100]. Aspirin taken daily for 5 years or longer at a minimal dose of 75 mg reduced both incidence and mortality due to CRC with the greatest preventive effect for cancers of the proximal colon. Importantly, aspirin reduced the incidence of CRC and benign polyps (adenomas) in the general population and individuals with a history of adenomas or CRC [100, 101]. In the general population, the CRC incidence was decreased by 26% (RR 0.74, 95% CI 0.57 to 0.97), while in patients with a history of adenomas or CRC, risk of adenoma recurrence was reduced by 21% [relative risk (RR) 0.79, 95% confidence interval (CI) 0.68 to 0.92]. In case-control studies, regular use of aspirin has also been documented to reduce risk of CRC in good agreement with results of the randomized trials [102].

The preventive role of aspirin has now been documented in multiple studies of other cancers [78, 103].

The assessment of short-term effects of aspirin from the analyses of 51 randomized controlled trials that were designed to evaluate the role of aspirin in the primary and secondary prevention of vascular events demonstrated that aspirin reduced the incidence of several cancers from 3 years onwards [104]. In addition to having a preventive role in cancer, depending on the duration of treatment, aspirin also improves the results of treatment among cancer patients [7, 17, 24].

Pooled data for eight randomized controlled trials demonstrated that daily aspirin reduced deaths due to cancer (OR 0.79, 95% CI 0.68 to 0.92) [24]. A 5 years follow-up (data assessed in seven trials) revealed a significant benefit from daily aspirin for all cancers (HR 0.66, 95% CI 0.50 to 0.87) with the best results observed for gastrointestinal cancers (HR 0.46, 95% CI 0.27 to 0.77). Interestingly, three trials that assessed the 20-year risk of cancer death also showed that patients who took aspirin had a lower risk of cancer-associated death (HR 0.78, 95% CI 0.70 to 0.87 and for gastrointestinal cancers HR 0.65, 95% CI 0.54 to 0.78). Taking into consideration all solid cancers, the benefits significantly increased with treatment duration of 7.5 years and longer. The subgroup analysis indicated that for esophageal, pancreatic, brain, and lung cancers, this time may be around 5 years. Another important conclusion by authors was that the effects of aspirin did not depend on dose (75 mg and over 75 mg) and did not differ in relation to gender and smoking status. The final result was that the absolute reduction in 20-year risk of cancer death was 7.08% (95% CI 2.42 to 11.74) in patients 65 years or older at randomization. The decreased risk of death among patients diagnosed with cancer during trials may have resulted from the lower frequency of distant metastases observed in the aspirin group *versus* control [105], particularly in patients with colorectal cancer (HR 0·26, 95% CI 0·11–0·57, *p* = 0·0008). Aspirin lessened the frequency of metastasis both at initial diagnosis and during follow-up in patients who did not present with metastasis initially.

At this point, data regarding breast cancer are inconclusive [17, 106]. A 10-year Women’s Health Study Trial with almost 40,000 women did not correlate aspirin use with decreased breast cancer incidence [26], while other meta-analyses have demonstrated less development of breast cancer (9–30%) among women who have been taking aspirin [107, 108]. The differences in results may be associated with the length of time aspirin is taken [17] Shiao]. A prospective observational study of 4164 women with breast cancer showed that decreased risk of distant recurrence and breast cancer death is observed when aspirin is taken at least 12 months [7]. The newest retrospective analysis of 222 stage II and III triple-negative breast cancer (TNBC) patients demonstrates improvement in 5-year DFS (80.1 *vs* 62.6%, *p* = 0.04) and 5-year DMR (9.0 *vs* 31.6%, *p* = 0.007) in the aspirin group compared with the control group. Intriguingly, aspirin also decreased the rate of central nervous system (CNS) metastases.

The benefits of an aspirin regimen were also documented for lowering prostate cancer incidence [109–112] and for prostate cancer patients, especially in the high-risk group [4, 113]. The study, comprising nearly 6000 patients after prostatectomy or radiotherapy who received different anticoagulants (AC), showed that aspirin use was associated with lower prostate cancer-specific mortality (PCSM) compared with the non-AC group (adjusted hazard ratio 0.43; 95% CI 0.21 to 0.87; *P* = 0.02) [4]. Disease recurrence and bone metastasis were also significantly lower in the AC group.

The prevention of carcinogenesis in cell line models and improvement of recurrence-free survival and overall survival (52.8 *vs* 47.9%; *p* = 0.021 and 80.3 *vs* 65.4%; *p* < 0.001, respectively) were observed among HBV-related hepatocellular carcinoma (HCC) patients after liver resection [20].

Aspirin assessment in cancer patients summarizes Table 2.

**Table 2:** Aspirin assessment in cancer patients

Cancer	Study	Results
Colorectal (CRC)	Rothel 2010 [101] Analysis of randomized trials Cooper 2010 [100] Analysis of randomized trials	Aspirin taken daily for 5 years and longer at the dose of at least 75 mg reduced both incidence and mortality due to CRC. Decreased risk of adenomas in CRC patients.
Prostate cancer	Choe [4] *n* = 6000 A multi-institutional registry	Lower prostate cancer-specific mortality (3 v 8%). Increased risk of upper gastrointestinal bleeding
Hepatocellular carcinoma	Lee 2016 [20] Retrospective analysis, *n* = 442	Improvement of recurrence-free survival and overall survival, reduced the risk of HCC recurrence and overall mortality
Breast cancer	Holmes 2010 [7] Prospective observational study, *n* = 4164	Decreased risk of distant recurrence and breast cancer death
Gastrointestinal, esophageal, pancreatic, brain, and lung cancers	Rothel 2011 [24] Analysis of eight randomized trials	Reduced deaths due to cancer

The recommendations on the preventive use of aspirin are summarized on the official website of the US preventive services task force (Table 3).

**Table 3:** Recommendations on the preventive use of aspirin of the US preventive services task force

Population	Recommendation
Adults aged 50 to 59 years with a ≥10% 10-year CVD risk	Initiating low-dose aspirin use for the primary prevention of cardiovascular disease (CVD) and **colorectal cancer (CRC)** in adults aged 50 to 59 years who have a 10% or greater 10-year CVD risk, are not at increased risk for bleeding, have a life expectancy of at least 10 years, and are willing to take low-dose aspirin daily for at least 10 years.
Adults aged 60 to 69 years with a ≥10% 10-year CVD risk	The decision to initiate low-dose aspirin use for the primary prevention of CVD and **CRC** in adults aged 60 to 69 years who have a 10% or greater 10-year CVD risk should be an individual one. Persons who are not at increased risk for bleeding, have a life expectancy of at least 10 years, and are willing to take low-dose aspirin daily for at least 10 years are more likely to benefit. Persons who place a higher value on the potential benefits than the potential harms may choose to initiate low-dose aspirin.
Adults younger than 50 years	The current evidence is insufficient to assess the balance of benefits and harms of initiating aspirin use for the primary prevention of CVD and **CRC** in adults younger than 50 years.
Adults aged 70 years or older	The current evidence is insufficient to assess the balance of benefits and harms of initiating aspirin use for the primary prevention of CVD and **CRC** in adults aged 70 years or older.

#### Lipoxygenase inhibitors

The enhanced expression of 5-LOX and 12-lipoxygenase (12-LOX) is associated with carcinoma progression and invasion [114, 115]. Increased expression of 5-LOX was investigated in adenomatous colon polyps and cancer compared with normal colonic mucosa, while 12-LOX expression was higher in more advanced stages of prostate cancer and in high-grade tumors [116].

Overexpression of the platelet 12-LOX in PC-3 prostate cancer cells results in 12(S)-HETE production which leads to the activation of transcription factors such as NF-κB resulting in increased MMP9 expression and in hypoxia inducible factor-1alpha (HIF-1alpha)-mediated in VEGF expression [71, 72, 117]. There is also evidence that platelet 12-LOX-mediated AA metabolism is responsible for radioresistance of prostate cancer cells and metastatic potential of melanoma cells [118]. The experimental *in vitro* and *in vivo* studies demonstrated the antiproliferative potential of LOX-inhibitors toward cancer cells [119]. The 5-LOX inhibitor zileuton decreased tumor growth of HT29 and LoVo human colon cancer cells in a heterotopic xenograft model in athymic mice [115]. The LOX inhibitors (nonselective nordihydroguaiaretic acid, 5-LOX inhibitor Rev-5901, and the 12-LOX inhibitor baicalein) suppressed proliferation of pancreatic cancer cells and decreased survival of prostate cancer cell line [120]. The newest approach is based on even dual inhibition of COX and LOX activity which leads to reduced proliferation and decreased tumor burden as well as cell migration and invasion [68, 117].

## ADP receptors

### ADP receptor P2Y12 antagonists, thienopyridines, and pyrimides

The platelet adenosine diphosphate (ADP) receptor P2Y12 is essential for thrombus formation by amplifying the hemostatic responses activated by a variety of platelet agonists. It stabilizes platelet aggregation and is involved in granule secretion as well as integrin activation. Apart from its role in hemostasis, the P2Y12 receptor has been shown to influence multiple mechanisms leading to cancer progression mainly by regulating tumor cell/platelet interaction and angiogenesis [121, 122]. There is also evidence that P2Y12 receptors mediate tumor cell transendothelial migration [123]. P2Y12 inhibition hampers LLC cells from influencing platelet shape and induces release of active TGFβ1 that disrupts platelet-mediated EMT-like transformation of the LLC. Similar effects were documented for melanoma cells [121].

Thienopyridines are antiplatelet drugs that inhibit the platelet adenosine diphosphate (ADP) receptor, P2Y12, and in that way inhibit ADP-mediated platelet activation and aggregation. The *in vitro* studies demonstrated the anticancer activity of thienopyridines in multiple cancer cell lines and cancer murine models (e.g. ovarian, breast, hepatocellular cancer, melanoma) [7, 85, 124]. The mechanisms of curtailing the invasive capacity of cancer cells were associated with suppression of platelet aggregation and adhesion, and decreased potential for platelets to create complexes with tumor cells [7, 32, 125]. Moreover, P2Y12 inhibitors influenced platelet proangiogenic potential in cancer patients [7]. Vascular endothelial growth factor and endostatin are the most vital protein modulators of angiogenesis released from platelets by the ADP-dependant pathway [1]. Cangrelor, the *in vitro* equivalent of clopidogrel, decreased secretion of VEGF, TSP1, and TGF-β1 by platelets to a greater extent when coming from cancer patients than from healthy controls. Thienopyridine SR 25989, an enantiomer of the anti-aggregant clopidogrel, inhibited angiogenesis by increasing the expression of endogenous TSP1 and reduced lung metastasis in metastatic melanoma mouse model [126].

Within the thienopyridine family, the ADP receptor inhibitor ticlopidine exhibited inhibitory effects on pulmonary metastases of B16 melanoma and AH130 ascite hepatoma as well as spontaneous metastases of LLC [94, 127]. The reversible P2Y12 inhibitor ticagrelor, recommended for the prevention of cardiovascular and cerebrovascular events, exerted an inhibitory metastatic effect in intravenous and intrasplenic melanoma and breast cancer mouse models [124]. The reduction of lung, liver, and bone marrow metastasis rate was by 55–84, 86, and 87%, respectively. The decreased interactions between tumor cells/platelets were the consequence of disrupted GPIIbIIIa expression. However, these findings may be context dependent [128].

Additionally, the anticancer/antimetastatic activity of thienopyridines may be associated with their inhibiting action on the interactions between cancer cell-derived tissue factor-positive microparticles (TMP), fibrinogen, and activated platelets [129, 130]. Study with human pancreatic adenocarcinoma cell lines showed that tumor-derived TMPs may activate platelets *in vitro* and in mice leading to thrombosis. The administration of clopidogrel inhibited thrombus generation and diminished tumor size in a mouse model by preventing the P-selectin and integrin-dependent accumulation of cancer cell-derived microparticles at the site of thrombosis.

The influence of antiplatelet treatment based on clopidogrel and aspirin on number of CTC numbers was investigated in a randomized phase II trial in breast cancer patients [131]. Unfortunately, there was no difference in the number of patients with detectable CTCs between the clopidogrel/aspirin and control groups suggesting that such an approach has no treatment effect.

Aspirin and thienopyridine are an established Dual Antiplatelet Therapy (DAPT) for reduction of stent thrombosis and myocardial infarction after coronary stenting. The alarming data that such an approach may be associated with increased cancer incidence forced re-analysis of the main trials from this point of view [29–31]. The adverse effects of antiplatelet therapy were first observed during the TRITON trial in which prasugrel *versus* clopidogrel was assessed in patients with acute coronary syndromes and for vorapaxar treatment in the TRACER trial [30]. Ticagrelor treatment (reversibly binding oral P2Y12 receptor blocker) assessed in the PEGASUS trial was also associated with significant excess in cancer death [132]. DAPT therapy from a 30-month trial with prasugrel and clopidogrel provided abundant data [30]. This trial revealed that a long-term antiplatelet approach is associated with cancer development that contributes to about half of noncardiovascular deaths (NCVD). The outcomes of the analysis indicated that cancer risk that reflects a class effect and is not drug-specific occurs after 24 months of extended antiplatelet therapy. It is suggested that extensive periods of antiplatelet therapy render platelets unable to aggregate with tumor cells and restrict them locally *in situ* [31]. For this reason, antiplatelet therapy with thienopyridines is recommended not to exceed 2 years. Such an approach reduces cancer risk, while most vascular benefits are still preserved [30]. Likewise, triple therapy aggressive regimens, lasting more than 1 year, are not recommended and intake of additional NSAIDS in combination with these regimens must be considered carefully for cardiovascular risk [133].

Importantly, there are some controversies in the construction of DAPT trial highlighted by FDA (Food and Drug Agency) which might have affected the final results [31]. There was imbalance between groups enrolled in the study. Namely, 22 more patients were randomly assigned to the thienopyridine group than to the placebo group leading to a difference in the number of patients in whom cancer had been diagnosed before enrollment (8 *vs* 1). Moreover, the rate of diagnosis of cancer did not differ significantly after randomization, even though there were more cancer-related deaths among patients treated with continued thienopyridine than among those who received placebo. Apart from that there is a lack of precise information about dosages for prasugrel and aspirin and the follow-up details. DAPT trial did not also describe how the follow-up statistics count deaths, allowing for about 5% data being missing. The Agency suspect that the way the enrolled patients were selected for randomization could, in turn, affect the cancer risks. Taking into consideration that the DAPT trial was a large, randomized trial, the initial risks should have been equal in both arms.

The doubts are enhanced by the fact that there are other data not confirming these alarming findings. In direct contrast to derived from the aforementioned clinical trials, a population-based cohort study of 10,359 colorectal, 17,889 breast, and 13,155 prostate cancer patients showed no evidence of an increased cancer-specific mortality among clopidogrel users [32]. The retrospective analysis among patients with hepatocellular cancer after liver resection showed that use of aspirin or clopidogrel was associated with better recurrence-free survival and overall survival among patients with HBV-related HCC [20]. Moreover, the analysis of survival after solid cancer (SASC) rates in patients enrolled in antithrombotic trials showed that survival in these groups of patients does not differ from Surveillance, Epidemiology, and End Result (SEER) or World Health Organization averages [31].

### ADP scavenger apyrase

ADP is a marker of platelet activation stored in dense granules that interacts with 2 P2Y receptors, the Gq coupled P2Y(1) receptor, and the Gi-coupled P2Y(12) receptor leading to platelet shape change, aggregation, and release of TxA2 [134]. ADP is involved in TCIPA. Very early studies showed that two ADP-scavenging agents, apyrase (10 U/ml) and creatine phosphate (CP) (5 mM)/creatine phosphokinase (CPK) (5 U/ml), completely inhibited TCIPA induced by B16-F10 melanoma and lung cancer cell lines [60]. Recent experiments were unable to confirm the inhibitory effect of apyrase on platelet aggregation and secretion [135]. Perhaps the inhibitory potential of ADP scavenger apyrase alone is too weak, as the novel soluble apyrase/ADPase, APT102, given together with aspirin, but not either drug alone, did inhibit TCIPA and lead to significant decreases in breast cancer and melanoma bone metastases in mouse models. Additionally, these effects were accompanied by fewer bleeding complications than observed with GPIIaIIIb inhibitors [136].

## Integrins

Integrins are transmembrane receptors composed of α- and β subunits [eprenbeck]. Their expression on platelets increases after platelet activation, which promotes integrin interactions with extracellular matrix (ECM) proteins [1, 2].

### Integrin αIIbβ3 (glycoprotein IIb/IIIa, GPIIb/IIIa) antagonists

The main glycoprotein on the surface of platelets and the key factor in carcinogenesis is αIIbβ3-Integrin (GP IIb/IIIa). GP IIb/IIIa contains an arginine-glycine-aspartic acid (RGD) sequence and functions as a receptor for fibrinogen and numerous other adhesion proteins [137]. GP IIb/IIIa regulates platelet aggregation and granule secretion as well as interaction between platelets, ECs, extracellular matrix components (ECM), and tumor cells resulting in platelet adhesion to these structures [138–140]. Thus, targeting the GP IIb/IIIa and subsequent impairment of platelet function within the tumor microenvironment provides a therapeutic option to inhibit metastasis.

The role of GP IIb/IIIa in TCIPA was first documented in 1987 by preincubation of human platelets with antibodies to platelet glycoprotein Ib and the IIb/IIIa complex [141]. The inhibitory action of monoclonal antibody against GPIIb/IIIa, 10E5, on pulmonary metastasis was subsequently determined using *in vivo* models the following year [142]. Since then, many inhibitors of the GP have been shown to prevent ECs, ECM, and cancer cell-platelet interactions, e.g., RGDS peptide, tirofiban (nonpeptide mimetic of the RGD sequence), eptifibatide (cyclic heptapeptide based on the KGD amino acid sequence), and abciximab (monoclonal antibody 7E3 Fab fragment) [143–145]. These three inhibitors (abciximab, eptifibatide, and tirofiban) are currently approved for human use in cardiovascular diseases [146].

Glycoprotein IIb/IIIa antagonists exert a strong anti-platelet effect by inhibition of the final pathway of platelet aggregation and reduce platelet adherence to tumor cells, ECM, and ECs. GP IIb/IIIa inhibition resulted in TCIPA blockade in MCF-7 breast cancer, human cervical carcinoma (HeLa), Lewis Lung carcinoma (LL2), and B16-F10 mouse melanoma cell lines resulting in decreased hematogenous metastasis to the lung in a mouse model [147–150]. Inhibition of TCIPA was also documented for snake venom Arg-Gly-Asp-containing peptides (e.g. triflavin, rhodostomin, trigramin) against osteosarcoma cells, hepatoma cells, and several adenocarcinoma cells (e.g. prostate, breast) [151–153]. These drugs exerted their effects by binding to the fibrinogen receptor associated with GP IIb/IIIa complex on the platelet surface.

More recent *in vitro* findings have documented the pivotal role of platelet GP IIb/IIIa in the development of bone metastasis by mediating tumor cell/host stroma interactions [154–156]. As many as 74% of mice expressing beta3 integrin developed osteolytic bone metastasis after injection of B16 melanoma cells intracardialy, while only 4% of mice depleted of beta3 integrin demonstrated bone lesions [154]. Rhodostomin, a disintigrin, strongly inhibited the adhesion, migration, and invasion of tumor cells in bone extracellular matrices [49].

It was demonstrated that a novel humanized single-chain antibody (scFv Ab; A11) against integrin GPIIIa49–66 could induce platelet lysis within the tumor environment [157]. This compound effectively inhibited multiple interactions between tumor and host cells with the end result being blocked development of pulmonary metastases in the LLC metastatic model. There is also an orally administered non-peptide small molecular peptidomimetic inhibitor of GPIIb-IIIa (XV454). It has been shown to reduce TCIPA *in vivo* and experimental metastasis in the Lewis lung carcinoma (LL2) mouse model [158].

GPIIb-IIIa inhibitors may also affect angiogenesis by reducing the release of VEGF from platelets as well as decreasing platelet/tumor cell complexes adherence to ECs and ECM an important step in angiogenesis [138, 139]. Additionally, the monoclonal antibody 7E3 F(ab’)2 reduced platelet-stimulated sprouting of ECs and tube formation [159, 160].

New studies have focused on the integrin β subunit plexin-semaphorin-integrin (PSI) domain that has endogenous thiol isomerase activity and is potentially a novel target for anti-platelet therapy [161]. The monoclonal antibodies directed to the PSI domain reduce the interaction of αIIbβ3-integrin with fibrinogen as well as inhibit murine and human platelet aggregation *in vitro* and *ex vivo*. This study showed that the PSI domain is an important factor for integrin functions and cell-cell interactions, such that its inhibition should be investigated beginning with cancer cell lines.

The inhibitory potential toward platelet integrins is also exerted by holothurian glycosaminoglycan (hGAG), a sulfated polysaccharide extracted from the sea cucumber, *Holothuria leucospilota* Brandt [162]. It blocks interactions between breast cancer cells MDA-MB-231 and platelets by suppressing both mRNA and protein levels of β1 and β3 integrins, as well as MMP-2 and -9, while increasing the expression of the MMP inhibitor, tissue inhibitor of metalloproteinase (TIMP)-1. hGAG reduced thrombin-induced platelet activation and aggregation, and disrupted platelet adhesion to cancer cells and fibrinogen to ultimately attenuate platelet-cancer cell complex formation.

Although results coming from experimental studies are promising, there are no strong clinical data for use of β3 integrin blockers in cancer patients.

### Integrin α2β1

The role of platelet α2β1integrin in cancer is much less documented than GPIIbIIIa. However, there are some data that support its role in pancreatic cancer cell adhesion to ECM [163]. Moreover, recent findings demonstrated that β1 integrin-dependant interactions within the tumor microenvironment may modify tumor response to radiation and chemotherapy [164]. Therefore, targeting β1 integrin is also a promising anticancer approach.

## Glycoproteins

The receptor complex, GP1b/IX/V and platelet collagen receptor, GPVI plays a predominant role in platelet adhesion and activation in the blood stream by interactions with their ligands, von Willebrand factor (VWF) and collagen, respectively, resulting in platelet capture and rolling at the site of vascular injury [165]. Inhibition of platelet GP1b/IX/V or GPVI receptors has been shown to reduce TCIPA and attenuate metastases [166–169]. GPVI-deficient mice developed 50% less visible metastatic tumor foci in the lung as compared with control mice after injection of LLC (D121) or melanoma (B16 F10.1) cell lines. Intriguingly, the size of subcutaneously implanted tumor cells did not change in relation to presence or absence of platelet GPVI.

However, others have observed that inhibition of GPIbα by monovalent, monoclonal antibodies strongly increased lung metastasis in melanoma animal models [170].

## P-selectin inhibitors

There are three selectins, P-, L-, and E-selectin, which belong to the C-type lectins that recognize glycans [165]. The most important platelet-membrane integrin for regulating platelet interactions with mononuclear cells and cancer cells is P-selectin that is expressed by both platelets and ECs [1, 3]. P-selectin is implicated in blood coagulation by mediating fibrin generation. Increased expression of P- and L-selectins (expressed on leukocytes) is observed within the metastatic microenvironment of tumor cells, thus regulating thrombi formation [171]. Experimental metastasis models demonstrated that both P- and L-selectin-depleted mice had improved survival [172]. Heparin as well as other compounds (e.g. dermatan sulfates) inhibited P-selectin-based platelet interactions with carcinoma cell-surface mucin ligands resulting in reduced adherence of tumor cells to platelets and decreased metastatic dissemination [173–175]_._ The observed effects were not associated with the anticoagulant potential of heparin, but by its ability to behave as a P-selectin ligand to impair interactions between P-selectin and its cognate ligands [172].

## Thrombin inhibitors

Tissue factor expressed by multiple cancer cells triggers local generation of thrombin that is a known potent platelet agonist [3, 13]. Thrombin-induced platelet activation leads to secretion and release of TxA2 and proangiogenic molecules, enhanced expression of P-selectin, CD40 ligand, and integrins on platelet membrane, and increased release of fibronectin and VWF from platelet onto their surface. These thrombin-mediated effects promote primary tumor growth, chemotaxis, migration, intravasation, and angiogenesis thereby facilitating prometastatic adhesion events [3, 176]. Additionally, platelets coated with thrombin survive longer, providing a surface for cancer cells for adherence [1]. Thrombin triggers its effects mainly by PAR-1 and PAR-4 receptors (described later) [3]. Thrombin-induced platelet activation may be associated in part with ADP receptors, mainly P2Y(12) [177]. The inhibition of P2Y(12) attenuated the thrombin-mediated response in blood received from healthy individuals. The inhibition of thrombin-associated effects was mostly observed at thrombin concentrations that caused complete PAR-1 proteolysis and secretion of all ADP. The thrombin-dependent response was inhibited by 70–86% [177].

Over the last several decades, numerous studies documented the prometastatic potential of thrombin [reviewed in 3, 13]. Pretreatment of cancer cells with thrombin or preinfusion of thrombin into mice increased tumor size, enhanced invasive potential of several types of cancer cells, caused 4–413-fold increase in the number of experimental lung metastases, and decreased survival rates [149, 178–185]. In contrast, thrombin inhibition by dansylarginine *N*-(3-ethyl-1,5-pentanediyl) amide blocked TCIPA of colon cancer [186], while administering bivalirudin or oral reversible thrombostatin FM19 decreased prostate cancer cell invasion as well as tumor growth and angiogenesis in mouse model [187].

### Hirudin

Hirudin, a specific inhibitor of thrombin, diminishes invasive and metastatic potential of thrombin [185]. It decreased by almost half the amount of platelet-derived VEGF released at the site of the thrombus formation and inhibited metastasis in several experimental models [173, 174]. Hirudin inhibited TCIPA in non-small-cell lung cancer cell lines (NSCLC) and in SCLC, but in the last case, blocking TCIPA required addition of the ADP scavenger apyrase with hirudin [60]. Recombinant hirudin (rH) has also been shown to inhibit growth and exert anti-metastatic effects in several types of cancers through multiple mechanisms [185, 188]. Experimental studies with laryngeal carcinoma cells (HEp-2 cells) demonstrated that rH inhibited the adhesion, migration, and invasion of the cancer cells. Another study indicated that co-treatment with rH and liposomal vinblastine exerts a more potent anti-metastasic effect against murine B16 melanoma tumor growth compared to chemotherapy alone [189]. However, hirudin is less intensively investigated in preclinical studies due to its inhibitory potential of thrombin-fibrinogen interactions.

### Heparin

Heparin is commonly used in cancer patients to prevent or treat thrombosis. The anticoagulant therapy also improves survival rates in these patients by an unknown mechanism. Inhibition of thrombin generation induced by low molecular weight heparins, LMWHs in cancer cells, depends mainly on the anti-Xa activity [190]. The most probable anticancer mechanisms mediated by heparin are inhibition of tumor cell/platelet/endothelial interactions to suppress cell invasion and angiogenesis [191].

There is evidence that the important antimetastatic potential of heparin and modified heparin (with heparanase inhibitory activity) is due to their influence on selectin-induced interactions between tumor and blood cells (leukocytes, platelets) as well as ECs, thus inhibiting cancer cell colonization in distant tissues [192, 193]. It has been shown that heparin-mediated P-selectin inhibition disrupted interactions of endogenous platelets with sialylated fucosylated mucins on CTCs and reduced tumor cell survival [194]. Experimental metastatic models with LMWHs demonstrated varying potential of these drugs (tinzaparin, nadroparin, dalteparin, or enoxaparin) for inhibiting P-selectin binding and attenuation of metastasis. Namely, tinzaparin and nadroparin were strong inhibitors of P-selectin functions, while fondaparinux, a synthetic heparinoid pentasaccharide, was unable to suppress this protein and decrease the metastasis rate in a murine model [165, 195]. A new and unique heparin sulfate from the bivalve mollusk *Nodipecten nodosus* has been shown to inhibit P-selectin-mediated colon cancer cell/platelet complex formation and to decrease lung metastasis [196].

Heparin may also attenuate cancer cell dissemination by altering platelet angiogenic potential *via* decreasing secretion of platelet-derived VEGF [197]. The other mechanism of heparins in inhibition of hematogenous but not lymphatic metastasis [198] may be associated with decreasing tumor-induced VWF fiber formation and diminished platelet aggregation the fibrous net.

### DTIs

There are four parenteral direct inhibitors of thrombin (DTIs) activity that received Food and Drug Administration (FDA) approval in North America: lepirudin, desirudin, bivalirudin, and argatroban [199]. Argatroban has been shown to decrease tumor migration as well as bone metastasis in mouse breast cancer models [200, 201]. Animal models provided promising data about dabigatran etexilate, which administered twice daily at a dose of 45 mg/kg over 4 weeks, and attenuated invasive and metastatic potential of malignant breast tumors [202]. Dabigatran etexilate decreased tumor volume by 50% and led to reduction of tumor cells in the blood and micrometastases in the liver by 50–60%. Importantly, it has been shown that dabigatran etexilate and low-dose cyclosphosphamide (CP) reciprocally potentiate their own effects [203]. Namely, mice being co-treated with both drugs presented significantly smaller mammary tumors and developed fewer lung metastases than mice treated with CP or dabigratran etexilate alone. Similar observations have been made for dabigatran administered with cisplatin or gemcitabine in ovarian or pancreatic cancer, respectively [204, 205]. Dabigatran co-treatment with low-dose cisplatin or gemcitabine in mice was more efficient in decreasing tumor growth than dabigatran or chemotherapeutic given as a single agent. Dabigatran also exerted antiangiogenic potential by decreasing levels of Twist and GRO-α proteins as well as impeding vascular tube formation induced by thrombin in breast and glioblastoma cell lines [206]. The other thrombin inhibitor, FM19, was also found to reduce angiogenesis in prostate tumor models [187]. Additionally, dabigatran etexilate prevented a tumor-induced increase in circulating TF(+) microparticles and decreased the amount of activated platelets by 40% demonstrating thrombin participation in intravascular events [203].

### Targeting thrombin generation

The factor Xa inhibitors apixaban, edoxaban, fondaparinux, and rivaroxaban inhibit thrombin generation and influence thrombin-mediated platelet activation. It has been shown that the histological type of cancer influences the antithrombotic activity of the thrombin inhibitors, and this should be taken into consideration when analyzing the results of experimental studies [207]. Fondaparinux has demonstrated inhibitory impact on platelet angiogenic potential. Decreased thrombin generation, which is the main activator of PAR-1 and PAR-4 present on the platelet surface, may disrupt PAR-mediated activation and in this way diminish angiogenic potential of cancer cells. The first clinical trial with thrombin inhibitor (Rivaroxaban) as an anti-cancer agent is now being established [208]. The efficacy of thrombin inhibition preoperatively (TIP) in supression of tumor proliferation will be investigated in estrogen receptor negative early breast cancer patients. The reduction in tumor Ki67 from pre-treatment to 14 days post-treatment initiation will be controlled.

The new research has provided evidence that V600EB-Raf signaling is involved in thrombin generation in metastatic melanoma *via* increased expression of TF on cell membranes [209]. BRAF is a component of the RAS–RAF–MEK–ERK signaling pathway that plays a critical role in cell proliferation, differentiation, and survival. Targeting V600EB-Raf-associated intracellular pathways and decreased thrombin generation may be another target for anticancer treatment.

Although clinical trials provided promising data that heparin treatment improved survival in cancer patients, it is still not recommended as anticancer approach [210].

## PARs inhibitors

Thrombin activates human platelets by triggering a family of G protein-coupled receptors, PARs, in a unique proteolytic mechanism. Thrombin opens the receptor active site by cleavage of a key extracellular domain of the N-terminus of the receptor. After cleavage, the newly formed amino-terminal peptide acts as a tethered ligand that binds to the receptor inducing intracellular signaling [3]. There are four PARs, PAR-1 to PAR-4, but human platelets express only high-affinity PAR-1 and low-affinity PAR-4 receptors that interact with different concentrations of activator [3]. Thrombin-induced cleavage of PAR-4 causes longer activation of Gα_q_ and sustained Ca^2+^ response, resulting in prolonged secondary signaling, compared to PAR-1, contributing to the late-phase of platelet aggregation. The thrombin-elicited platelet PAR activity also exerts mitogenic activity toward ECs and myocytes of vessels [3]. The evidence for a pivotal role of thrombin-mediated platelet PAR activation in cancer dissemination comes from studies performed on mice depleted of platelets, PAR-4, or fibrinogen—these animals presented significant reduction in experimental pulmonary metastasis [211].

Both receptors activate pleiotropic cellular effects, one of which is stimulation of angiogenesis. Previous findings that thrombin activation of PAR-1 leads to VEGF secretion from platelets, while activation of PAR-4 results in the release of an inhibitor of angiogenesis–endostatin, were recently disputed [212]. Namely, the activation of platelet with either PAR-1 or PAR-4 activated peptides resulted in significant VEGF secretion. Moreover, both platelet PAR-1 and PAR-4 activation promoted angiogenic activities of endothelial progenitor cells. However, PAR-1-mediated platelet releasate acted more potently than PAR-4-stimulated proteins in vasculogenesis [213].

PAR-1 and PAR-4 induced platelet activation leads to synthesis and release of TxA2 and 12(S)-HETE, which, when esterified to phosphatidylethanolamine, acquire unique functions in that context [214, 215].

Importantly, platelet PAR-1 may also be triggered by MMP-1, leading to Rho-GTP as well as MAPK signaling activation, resulting finally in platelet aggregation, and increased motility and cell proliferation [216]. The ProMMP-1 zymogen is converted to MMP-1 on the platelet surface with the presence of collagen fibrils. Inhibition of MMP-1-mediated PAR-1 signaling is efficient to block thrombosis in animal models, giving evidence that the collagen/MMP-1/PAR-1 pathway may stimulate platelet signaling events independent of thrombin. As PARs also stimulate the expression and release of 12(S)-HETE that upregulates MMP9 [72], it seems that bi-directional regulation of MMPs and PARs signaling exists.

PAR-1 also promotes expression of melanoma cell adhesion molecule MCAM/MUC18 (MUC18), a crucial marker of melanoma metastasis. It is of interest that PAR-1 activity increases expression of platelet-activating factor receptor (PAFR) and its ligand and, in this way, not only induces platelet aggregation but also increases MUC18 levels. The PAR1/PAFR/MUC18 pathway regulates melanoma cell adhesion to microvascular ECs and transendothelial migration, and finally enhances metastatic seeding in the lungs [217]. Upregulation of PAFR and its agonists in this context may also impact immune functions against cancer [218–222].

The pleiotropic mechanisms regulated by PARs make inhibitors of these receptors an exciting option for antiplatelet and anticancer treatment. Many compounds, such as RWJ-58259, SCH-79797, or RWJ-56110, were developed that target PAR-1 and exert strong antithrombotic effects *in vivo* [223–225]. Additionally, PAR-1 small-interfering RNA (siRNA) incorporated into neutral liposomes (1,2-dioleoyl-sn-glycero-3-phosphatidylcholine, DOPC) was assessed in experimental melanoma models. Mice treated with the PAR-1 siRNA-DOPC presented a decline in tumor growth, weight, and formation of metastatic lung colonies [226]. siRNA delivery also affected angiogenesis by reducing VEGF, Il-8, and MMP-2 expression levels, and blood vessel density. PAR-1 silencing downregulates expression of the adhesive protein MUC18, which also attenuates the metastatic phenotype of melanoma cells [226].

The first PAR-1 antagonist, vorapaxar, was recently approved for use as an antiplatelet agent to reduce the frequency of ischemic events [227]. However, vorapaxar and other PAR-1 inhibitors may cause serious adverse effects associated with bleeding events or cause increased cancer death (data derived from TRACER trial) [31]. These results have led to recent studies to determine the specific antiplatelet effects of inhibiting PAR-4 function. A rabbit polyclonal antibody against the thrombin cleavage site of PAR-4 alone (without PAR-1 inhibition) effectively inhibited thrombin-induced cellular activities like elevation of cytosolic calcium levels and consequent phosphatidylserine exposure. Unfortunately, and important with respect to anticancer treatment, PAR-4 inhibition did not suppress integrin αIIbβ3 activation nor did it decrease α-granule secretion and platelet aggregation—the mechanisms strongly contributing to cancer progression. Thus, no clear progress in the investigation of antiplatelet/anticancer therapy was made.

Although PARs-targeted drugs have been implemented in the treatment of cardiovascular disease (vorapaxar, Zontivity) in cancer disease, these have not made it to clinic for treatment of cancer [228]. As platelet activation is mediated by multiple molecular signaling pathways, inhibition of only one may not be effective. A study showing that both ADP inhibitor and antagonists of PAR-1 (SCH 19197), or its activator kallikrein, should have been used to inhibit platelet activation suggested that bi-modal inhibition may need to be applied to achieve clinical benefit [229].

## CLEC-2 inhibitors and podoplanin-targeting cancer therapy

There is a newly discovered surface receptor on platelets, C-type lectin-like receptor 2 (CLEC-2), that is activated by the mucin-type sialoglycoprotein podoplanin (aggrus), which is a known platelet aggregation-inducing factor present on multiple tumor cells (squamous cell carcinoma, mesothelioma, glioblastoma, sarcoma) and lymphatic endothelial cells [230–232]. Physiologically, podoplanin takes part in lymphatic vessel development and thrombus formation but in the presence of tumor cells may promote tumor growth and hematogenous tumor metastasis by inducing secretion of growth factors and inducing tumor emboli formation in the microvasculature [232]. The disialyl-core1-attached glycopeptide as well as the stereostructure of the podoplanin protein was found to be critical for the CLEC-2-binding activity of podoplanin [231]. There are platelet aggregation-inducing domains on human podoplanin, PLAG4 (81-EDLPT-85), and PLAG3 that contribute to the binding of platelet CLEC-2. Studies showed that simultaneous inhibition of PLAG3/4 is indispensable for complete suppression of podoplanin-mediated procancer activity. CLEC-2 induces intraplatelet signaling in conjunction with a single YxxL motif in its cytoplasmic tail, Src and Syk kinases, and phospholipase Cγ2 [232].

B16F10 melanoma cells co-cultured with wild-type platelets, but not with CLEC-2-deficient platelets inhibited by anti-mouse CLEC-2 monoclonal antibody 2A2B10, demonstrated increased proliferation. Hematogenous metastases were also significantly inhibited in CLEC-2-depleted mice giving the rationale for targeted inhibition of CLEC-2 as a new strategy for preventing hematogenous tumor metastasis. The monoclonal antibody NZ-1 directed against podoplanin disrupted both the podoplanin-CLEC-2 interaction and podoplanin-induced pulmonary metastasis [231]. Another non-cytotoxic 5-nitrobenzoate compound, 2CP, specifically inhibited the podoplanin-CLEC-2 interaction and TCIPA [233] resulting in anti-cancer metastatic activity *in vivo* in the experimental animal model. Importantly, inhibition of CLEC-2 receptor potentiated the therapeutic efficacy of cisplatin without increasing the risk of bleeding. 2CP directly blocks the region for podoplanin on the CLEC-2 receptor, affects Akt1/PDK1 and PKCμ signaling pathways, and by this mechanism inhibits platelet activation. The fact that absence or reduction of CLEC-2 receptor activity on platelets decreases the hematogeneous metastasis rate without significantly increasing bleeding tendency indicates that CLEC-2 may be a good target for anti-platelet/anti-metastatic drugs.

High expression of podoplanin was demonstrated in primary brain tumors and contributed to platelet aggregation and hypercoagulability state, suggesting the potential antitumor role of podoplanin inhibitors in glioblastoma [234].

## Microbial proteases

Platelets are now regarded as components of the immune system that are able, similar to leukocytes, to tether microbial pathogens *via* Toll-like receptors (TLRs) [11]. It is estimated that over 300 proteins and molecules (e.g. CD40, CD154) may be released to the vasculature due to platelet activation elicited by direct contact with pathogens. Platelets may also adhere to endothelial walls and leukocytes in the place of inflammation forming thrombi with microbial pathogens, and this way separate them in the blood. Considering the fact that tumors also promote inflammation, the influence of platelets on immune system is meaningful.

On the other hand, the role of microbial proteases in inflammation and carcinogenesis has now been widely investigated [reviewed in 3, 239]. It was demonstrated that arginine-specific gingipains from *Porphyromonas gingivalis* may lead to platelet activation through PAR cleavage and thus cause platelet activation and aggregation [235]. Additionally, it has been shown that the same bacteria may secrete proteases that activate PAR and proteases with inhibitory potential. *Pseudomonas aeruginosa* secretes protease LepA, which is able to activate PARs-1, -2, and 4 leading to stimulation of the NF-κB pathway and the elastolytic metalloprotease that inactivates PAR-2 by proteolysis of the extracellular part of this receptor [236, 237]. Antiplatelet activity has been described for a serine protease with fibrin(ogen)olytic properties named Bacethrombase that is produced by *Bacillus cereus* strain FF01 [238]. Bacethrombase inhibited ADP-induced platelet aggregation in a dose-dependent manner (Table 4).

**Table 4:** Platelet receptors implicated in tumor progression and their inhibitors

Receptor	Inhibitor, drug	*In vitro* effect	Animal models
ADP receptor P2Y12	SR 25989, cangrelor, clopidogrel, ticlopidine, ticagrelor	Inhibition of platelets aggregation, adhesion and complexes with tumor cells and suppression of angiogenesis	Reduction of lung, liver and bone marrow metastasis
GP IIb/IIIa	Tirofiban, eptifibatide, abciximab Rhodostomin A11 hGAG Inhibitor of PSI domain	Inhibition of platelets aggregation, adhesion, angiogenesis Reduction of TCIPA Inhibition of the adhesion, migration and invasion of tumor cells in bone extracellular matrice Platelet lysis within tumor environment Inhibition of platelet/tumor cells complexes Inhibition of platelet aggregation	Inhibition of metastasis on Lewis lung carcinoma mouse model
GP1b/IX/V, GPVI	Monovalent Fab fragments	Reduction of TCIPA	Inhibition of metastases
P-selectin	Heparin, dermatan sulfates	Inhibition of interaction between platelet and tumor cells	Inhibition of metastases
CD 151 [Huang 2016]	CD 151-directed antibody	Inhibition of angiogenesis	–
PAR-1, PAR-4	RWJ- -58,259, SCH-79797 or RWJ-56110 siRNA	Inhibition of interaction between platelet and tumor cells, inhibition of angiogenesis	Inhibition of tumor growth, weight, and metastasis
CLEC-2 and podoplanin	2A2B10, NZ-1	Inhibition of TCIPA	Inhibition of pulmonary metastasis

*ADP* adenosine diphosphate, *CLEC-2* C-type lectin-like receptor-2, *hGAG* holothurian glycosaminoglycan, *PAR* protease-activated receptors, *PSI* plexin-semaphorin-integrin

## Microparticles inhibitors

Platelets are known to release microparticles (MP) containing compounds that promote invasiveness of cancer cells and metastatic potential [239]. It is well recognized that glioblastoma increases risk of thrombotic events that activate platelets and may induce MP-mediated interactions between tumor cells, platelets, and the vasculature. Platelet-derived MPs are responsible for immunosuppression in the glioma microenvironment inducing glioma invasiveness and neovascularization [240]. Platelets may take up MPs of tumor cells and carry EGFRvIII transcript. Platelet MPs also contain microRNAs that have been shown to be released after platelet activation with thrombin. It has been demonstrated that MPs laden with miRNA may be internalized by human umbilical vein endothelial cells (HUVEC). MicroRNA in complex with Argonaute 2 (Ago2), Ago2 miR-223, regulates transcription and translation of endothelial cell genes, e.g., promoting advanced glycation end product-induced vascular endothelial cell apoptosis *via* targeting insulin-like growth factor 1 receptor [241]. In that case, platelets serve as carriers of genetic information between different types of cells.

Several compounds that interfere with MP-mediated intracellular actions, e.g., vaccines or immunomodulators, are now being investigated [reviewed in 243].

## Other targets and inhibitors

### CD151 inhibitors

The tetraspanin CD151, primarily identified as a molecular organizer of interacting proteins (integrins, growth factors, MMPs) into tetraspanin-enriched microdomains, is also involved in tumor progression and is thus being seriously considered as a potential anticancer target [160, 242]. There is evidence that CD151 regulates tumor cell adhesion, migration and invasion/intravasation, and dissemination. This protein is also engaged in angiogenesis. Reportedly, platelet-expressed CD151 is important for the enhancement of ECFC tube formation, as a CD151 antagonist attenuated this effect [160].

### β-Nitrostyrene derivatives

β-Nitrostyrene derivatives are promising in the antiplatelet/anticancer approach due to their wide influence on platelet functions [135]. Studies revealed that β-nitrostyrene derivatives (4-methylene-dioxy-β nitrostyrene, MNS and 4-O-benzoyl-3-methoxyl-β-nitrostyrene, BMNS) prevented not only TCIPA, P-selectin expression, and PDGF secretion but also platelet adhesion to cancer cells. Incubation of platelets, activated previously by tumor cells, with MNS and BMNS resulted in inhibition of initial interactions [135].

### Curcumin

Curcumin (diferuloylmethane) is a polyphenol derived from the *Curcuma longa* plant that may exert antiplatelet and anticancer effects by cell cycle regulation and proapoptotic activities against multiple cancers, e.g., of the liver, skin, pancreas, prostate, ovary, lung, and head/neck [243, 244]. The antimetastatic actions of curcumin result from inhibition of transcription factors (e.g. NF-κB), inflammatory cytokines (e.g. CXCL1, CXCL2, IL-6, IL-8), proteases (e.g. MMPs), multiple protein kinases (MAPKs), and/or regulation of miRNAs (e.g. miR21, miR181b) [244].

### AMP activator

Adenylate cyclase/cyclic AMP activation leads to platelet inhibition both *ex vivo* and *in vivo*. CME-1, a novel polysaccharide from the mycelia of *Cordyceps sinensis*, activated cAMP resulting in inhibition of intracellular ATP release, decreased intracellular [Ca(+2)] mobilization and phosphorylation of phospholipase C (PLCγ2) and protein kinase C (PKC), ultimately inhibiting platelet aggregation. Cyclin AMP activation also suppressed signaling mediated by thrombin and AA, such as phosphorylation of p38 MAPK, extracellular signal-regulated kinase 2, c-Jun N-terminal kinase 1, and Akt [245, 246].

The anticancer effect of CME-1 was demonstrated on B16-F10 murine melanoma cell lines [247]. CME-1 inhibited MAPK signaling, thereby disrupting tumor cell migration and dissemination. However, there is no information available about the anticancer effect of CME-1 against other malignancies and its inhibitory effect on platelet/tumor cell interactions.

### Hinokitol

Hinokitiol is a natural bioactive compound found in *Chamacyparis taiwanensis*, widely used in cosmetics and food for its antimicrobial potential. Some studies revealed its antiplatelet activity [248, 249]. Hinokitiol inhibited PLCγ2, PKC, MAPKs, and Akt in collagen-activated human platelets. Unfortunately, it did not exert antiplatelet activity induced by other agents like thrombin, AA, and ADP. However, taking into consideration the fact that hinokitol may inhibit B16-F10 melanoma cell migration, it is of interest whether some form of tumor cell/platelet interaction could be affected by this compound. Possible mechanisms of hinokitiol action on migration of highly metastatic melanoma cell line should be investigated with respect to its inhibitory effect toward platelets, e.g., as an antagonist of collagen GP VI, or a factor that reduces the expression of MMP-1 that may be produced by platelets [160, 249].

### Extract of Caulis Spatholobi

Ethanol extract of Caulis Spatholobi has been found to block TCIPA and breast cancer metastasis by disrupting the formation of tumor cell/platelet complexes in mouse models after mouse tail vein injection of 4T1 cells [250]. Decreased tumor metastasis development due to dissociation of the tumor cell/platelet complex improved survival in a mouse breast cancer model.

### Tyrosine kinase inhibitors

Tyrosine kinases are essential for platelet signaling so that inhibitors of TKs used in cancer patient treatment may affect platelet functions to increase bleeding risk [251]. Blood of renal cancer patients treated with sunitinib as well as from healthy donors were analyzed after preincubation with sunitinib. Treatment with sunitinib decreased platelet number and their activity.

### PKC inhibitors

PKCdelta plays a crucial role in transducing the invasion-promoting effects of platelets in breast cancer cells, and the specific inhibition of PKCdelta may be a strategy to decrease platelet-mediated cancer cell invasion [252].

### STS

Staurosporine (STS) is a microbial alkaloid and potent PKC inhibitor that has been shown to induce platelet apoptosis and thus has been suggested to be a promising anti-cancer drug [253]. The experimental results are controversial though, as enzastaurin, another PKC inhibitor with known antiproliferative and proapoptotic effects in a variety of cancers has been shown to potentiate platelet aggregation and VEGF secretion thereby counteracting its anticancer potential [254].

### NF-κB-mediated platelet signaling inhibitors

The inhibition of NF-κB transcription factor should also be considered as antiplatelet/anticancer treatment, as this factor regulates platelet activation even though platelets lack a nucleus. NF-κB-regulated events include IKKβ phosphorylation, IκBα degradation, and p65 phosphorylation [255]. Experiments with washed platelets revealed that sesamol activated cAMP-PKA signaling, followed by inhibition of the NFκB-PLC-PKC cascade, ultimately leading to inhibition of [Ca(2+)] mobilization and platelet aggregation [256]. NF-κB is a transcription factor that regulates many important processes responsible for cellular homeostasis. Studies proved that although platelets are anucleated cells, they also express NF-κB that may influence platelet functions in a non-genomic/transcriptional way.

### Calcium channel-blocking agents: nifedipine, diltiazem, verapamil, or amlodopine

Calcium channels are mobilized during platelet activation, and so their inhibitors were analyzed for antiplatelet potential [257]. Platelet-rich plasma (PRP) derived from normal subjects was incubated with one of four drugs: nifedipine, diltiazem, verapamil, or amlodopine. These agents administered in high concentration inhibited osteogenic sarcoma-induced platelet aggregation.

### Blockers of collagen binding sites and galectin-3 inhibitors

Platelets may induce COX-2 expression in HT29 colon carcinoma cells resulting in increased expression of PGE2, upregulation of cyclin B1, and expression of proteins involved in epithelial-mesenchymal transition (EMT). Galectin-3 is a member of a family of carbohydrate-binding proteins with a unique collagen-like domain that plays a role in platelet-cancer cell crosstalk through interaction with platelet collagen receptors, e.g., GPVI. Inhibition of platelet-cancer cell interaction by a novel antiplatelet drug revacept, which blocks collagen binding sites, prevented platelet-induced mRNA changes of EMT markers in colon carcinoma cells [258].

### PDGFR inhibitors

Thrombin-activated platelets secrete α-granules containing high levels of platelet-derived growth factor receptor (PDGFR). PDGFR influences tumor invasion by increasing CTC survival by maintaining CTCs in an epithelial-mesenchymal transition (EMT) state within the blood vessel [259]. PDGFR activity is documented in the pathogenesis of glioblastoma (GBM) [260]. There is evidence that insulin-mediated signaling (insulin receptor, IR/insulin growth-like factor receptor, IGF1R) may trigger resistance to PDGFR inhibition. Co-targeting IR/IGF1R and PDGFR decreased the number of resistant clones *in vitro*. The enhanced growth inhibition was observed in rhabdomyosarcoma cell lines when cells were treated with dual IGF-1R and PDGFR-β agents *in vitro* [261]. Furthermore, PDGFR tyrosine phosphorylation may be inhibited by Compound C resulting in decreased proliferation of A172 glioblastoma cells [262].

### Platelet aggregation inhibitors

Platelet aggregation inhibitors (ditazole, bencyclan, cyproheptadine, dipyridamole, pentoxyfilline) showed no significant influence on metastases formation in animal tumor models [reviewed in 1]. Mopidamole was demonstrated to inhibit significantly spontaneous lung metastasis in syngeneic Wilms’ tumor of the rat, the C1300-neuroblastoma of the mouse, the HM-Kim mammary carcinoma of the rat, and Lewis lung carcinoma of the mouse.

### Tamoxifen

There is also evidence that tamoxifen, a selective estrogen receptor modulator used for the treatment of breast cancer, and its metabolite 4-hydroxytamoxifen exert antitumor activity through inhibition of platelet-dependant angiogenesis and metastasis formation [263]. Tamoxifen-treated platelets released lower levels of proangiogenic factors like VEGF, angiogenin, CXCL1, CCL5, EGF, CXCL5, and PDGF-BB, resulting in decreased capillary tube formation and diminished endothelial migration. The laboratory tests were confirmed *in vivo* in cancer patients treated with tamoxifen. Their platelets secreted lower levels of VEGF compared with patients not being treated with tamoxifen, and these were characterized as having less angiogenic and metastatic potential.

## Conclusions

Evidence for the contribution of platelets to cancer progression is substantial and continues to mount. However, despite the discovery of new platelet surface receptors and molecular mechanisms involved in carcinogenesis, there is still an unmet need for effective antiplatelet/anticancer therapy. There is an established preventive role for aspirin in colorectal cancer, but there are no breakthroughs in treatment procedures. The majority of data come from laboratory studies, and numerous clinical questions remain unanswered. There is insufficient information on how specific types of cancer and advancement of disease progression depend on platelet function. Additionally, chronic administration of antiplatelet agents and thrombocytopenia relate to risk of bleeding complications or even increased cancer incidence that must be considered in clinical practice. There are still questions as to whether single or dual antiplatelet regimens should be applied to cancer patients, how long it is safe to block platelets, and whether antiplatelet treatment should be connected with classic chemotherapy. Laboratory studies have demonstrated superior results with the co-treatment approach. Although several doubts related to antiplatelet therapy as anticancer treatment exist, the experimental results justify continuing an effort to discover new drugs and anti-platelet approaches.
